# The impact of physical activity on psychological distress in young people: An analysis of longitudinal data from the Millennium Cohort Study using causal machine learning

**DOI:** 10.1002/jcv2.70035

**Published:** 2025-08-19

**Authors:** Lewis W. Paton, Noemi Kreif, Lauren M. E. Aylott, Philip Kerrigan, Clau Nader, Lina Gega, Paul A. Tiffin

**Affiliations:** ^1^ Hull York Medical School University of York York UK; ^2^ Department of Health Sciences University of York York UK; ^3^ Centre for Health Economics University of York York UK; ^4^ School of Pharmacy University of Washington Seattle Washington USA; ^5^ Tees, Esk & Wear Valleys NHS Foundation Trust Middlesbrough UK

**Keywords:** activity level, distress, longitudinal studies, machine learning

## Abstract

**Background:**

Physical activity is often associated with positive psychological wellbeing in adolescents, and there is evidence from randomised trials to support this. However, the effect sizes reported from intervention studies vary considerably. Moreover, physical activity is a ‘complex intervention’ and findings from trials may not generalise to naturalistic settings. Estimating causal effects from observational data is challenging, though provides an opportunity to add to the evidence base in this area. We used newer machine learning‐based causal inference methods to estimate (i) the causal effect of physical activity on psychological distress in young people (ii) variations in effects across subgroups (‘heterogeneous treatment effects’).

**Methods:**

Data from the Millennium Cohort Study were available for 9123 adolescents. Self‐reported days of moderate/vigorous activity was recorded at ages 14 and 17. Psychological distress was measured at age 17 using the Kessler‐6 scale. Directed acyclic graphs were co‐produced with a youth advisory group and individuals with lived experience to identify confounders. Average and heterogeneous treatment effects of physical activity at age 14 on psychological distress at age 17, as well as concurrently at age 17, were estimated using causal machine learning methods (targeted learning, causal forests).

**Results:**

We observed no overall impact of physical activity at age 14 on self‐reported distress scores at age 17 (0.11 points, −0.09 to 0.31, *p* = .28), although there was a modest impact of physical activity at 17 on concurrent distress (−0.39, −0.61 to −0.18, *p* < .001). Meeting government guidelines on physical activity levels benefited boys more than girls, but had relatively less positive impact on young people with special educational needs.

**Conclusions:**

Whilst no overall impact of physical activity levels on youth distress was observed, boys may particularly benefit from targeted interventions in this regard. Causal machine learning is a promising approach to rapidly generating evidence from observational data.

## Key Points

**What's Known**

- The potential impact of increased physical activity on mental health outcomes in young people is uncertain.

**What's New**

- In this study we did not observe any overall causal impact of increased physical activity levels at 14 on youth distress at 17 years.
- However, males gained relatively more benefit from increased physical activity levels than females, and those with special educational needs appeared to benefit less from increased physical activity levels.

**What's Relevant**

- Causal machine learning is a promising approach to rapidly gaining evidence from observational data.

## Graphical abstract

Using causal machine learning, we estimated the causal effect of meeting government guidelines on physical activity on psychological distress in young people. Whilst observing no overall impact, we did identify some groups who benefit relatively more from meeting the physical activity guidelines, including males. Causal machine learning is a promising approach to rapidly gaining evidence relevant to youth mental health policy from observational data.

## INTRODUCTION

Physical activity is often associated with positive mental health and wellbeing in young people. Meta‐analyses of randomised control trials (RCTs) of physical activity‐based interventions generally report positive effects on psychological outcomes in adolescents (Carter et al., 2021; Wang et al., 2022). The outcomes employed are sometimes related to mental health and at other times wellbeing; these are overlapping but, to some extent, distinct constructs. Specifically, the former is related to distressing experiences likely to be related to the probability of having a diagnosed psychiatric disorder. The latter generally relates to experiences of ‘hedonia’ (enjoyment) and 'eudaimonia’ (positive functioning and meaning in life) (Ryan & Deci, 2001).

The effect size estimates in these systematic reviews vary and also depend on the specific outcome (e.g., anxiety vs. mood). Moreover, one systematic review reported no overall effect for either anxiety or depression (Neill et al., 2020). Additionally, reported effect‐sizes are generally smaller in non‐clinical populations (Carter et al., 2016; Carter et al., 2021; Tymms et al., 2016). RCTs are also frequently performed in optimal circumstances (Zwarenstein & Treweek, 2009) and their findings may not generalise to naturalistic settings for other reasons (McCambridge et al., 2014). Furthermore, physical activity is a ‘complex intervention’ and multiple factors may mediate any mental health benefits (Craft & Perna, 2004).

Internationally, health ministries recommend adolescents should aim for an average of 1 h of moderate to vigorous physical activity per day (Department of Health and Human Services, 2018; Department of Health and Social Care, 2019). ‘Moderate’ activity is defined as that which ‘will cause children to get warmer and breathe harder and their hearts to beat faster, but they should still be able to carry a conversation’. Meanwhile, ‘vigorous’ activity is defined as that which causes children to breathe ‘much harder and their hearts to beat rapidly, making it more difficult to carry a conversation’. The evidence‐base for this policy is not established.

The impact of physical activity levels on adolescent psychological outcomes is also likely to be heterogeneous. Exercise can even become maladaptive, for example, in restrictive eating disorders (Schaumberg et al., 2023). In this regard, RCTs are usually only powered to estimate population averaged effect‐sizes. Thus, there is a need to evaluate the causal impact of exposure to physical activity in general populations and subpopulations from observational data.

Machine learning‐based methods to obtain estimates of causal effects from observational data are now available. While they still rely on a number of assumptions in order to draw causal inferences—including the assumption of no unmeasured confounding (Naimi & Whitcomb, 2023)—causal machine learning methods do confer some advantages over existing approaches, as they efficiently concentrate on only estimating the effect of interest, not other parameters in a model. Two such methods are *targeted learning* (Van der Laan & Rose, 2011) and *causal forests* (Wager & Athey, 2018). These have to date had limited application to mental health research.

The Millennium Cohort Study (MCS) (University College London, 2024) is a large ongoing, longitudinal UK‐birth cohort study which plausibly provides the data required to explore the impact of physical activity levels on young people's psychological distress. In this study the aims were to:

- Co‐develop, with a youth advisory group and lived experience experts, a theoretical model for the relationship between physical activity and youth mental health.
- Estimate the causal effect of meeting the recommended physical activity levels at 14 on youth mental health at age 17.
- Estimate how the causal impact of (ii) varies across different subgroups of young people.

Our findings could guide the development of targeted public health policy, as well as demonstrating the potential of causal machine learning to rapidly create such evidence from observational data.

## MATERIALS AND METHODS

### Ethical approval

This study was a secondary analysis of de‐identified data and therefore exempt from ethical approval. This was confirmed in writing by the Chair of the University of York's Department of Health Sciences Research Governance Committee.

### Data availability and management

The MCS recruited 18,827 nationally representative children born between September 2000 and January 2002 (Connelly & Platt, 2014). We used data from those who had completed MCS wave six (age 14) and wave seven (age 17) and had information on self‐rated psychological distress at age 17 (University College London, 2024). To avoid dependency within families we randomly sampled each family so data from only one child were included. The final analytic sample consisted of *N* = 9123 individuals. Amelia II in R was used to singly impute missing values using expectation‐maximising with bootstrapping (Honaker et al., 2011).

#### Outcome measure

Our outcome variable was psychological distress, at age 17, measured by the six item version of the self‐reported Kessler Psychological Distress Scale (K6) (Kessler et al., 2002). The K6 has been validated in adolescents (Ferro, 2019) and can be used as a screening tool for mental illness (Furukawa et al., 2003). We used total score (i.e., 0–24) at age 17, with higher scores indicating higher levels of psychological distress.

#### Exposure

Participants at age 14 reported how many days, in the last week, of moderate to vigorous physical activity they had done, options being: ‘not at all’, ‘1–2 days’, ‘3–4 days’, ‘5–6 days’ and ‘every day’. Those who reported five or more days of physical activity were deemed as meeting government guidelines (i.e., defined as the ‘exposure’), to allow for reporting (measurement) error. To evaluate the potential impact of varying the government guidance on physical activity we performed a sensitivity analysis. This varied the definition of the ‘exposure’, defining it as (i) any physical activity versus none, (ii) 3 days or less of physical activity versus 4 days or more, and (iii) every day versus not every day.

#### Confounders

To define the set of confounders to be used, we co‐produced a directed acyclic graph (DAG) with a youth advisory group and individuals with relevant lived experience (Rodrigues, Kreif, Lawrence‐Jones et al., 2022). A DAG represents the hypothetical causal relationship between variables. It helps define the confounders needed for the modelling process. The co‐production process is detailed in Supporting Information S1: Section A. This process identified 24 potential confounders used in the causal modelling process (see Section A: Figure S1, Table S1). We included only those available at age 14 to reduce the risk of incorrect temporal ordering. There were two exceptions—sexuality and religion—which were only available at age 17. However, these could be assumed not to be influenced by physical activity levels.

### Analysis

We initially estimated the average causal impact (also known as the *average ‘treatment’ effect*, or ATE) of meeting the government guideline on physical activity at 14 on subsequent psychological distress at 17 years. We used ‘targeted learning’—a causal machine learning method to estimate the average causal impact effect of the exposure over the population (Schuler & Rose, 2017). We used the *tmle* function from the *TMLE* package in R (Gruber & Laan, 2012).

We then estimated variations in this average causal impact (also known as the *conditional average ‘treatment’ effect*, or CATE), as it potentially varied across different subpopulations of young people, according to personal characteristics. We used ‘causal forests’ to do this (Wager & Athey, 2018) using the *grf* package in R (Tibshirani et al., 2022). We then used multivariable linear regression models to estimate statistically significant predictors of variations in the treatment effect. Further details of the modelling methods and machine learning processes are available in Supporting Information S1: Section B. Additionally, our ability to draw causal inferences using these methods relies on four assumptions: *counterfactual consistency, no interference, conditional exchangeability* and *positivity* (Naimi & Whitcomb, 2023). Further details on these assumptions are available in Supporting Information S1: Section B.

Data cleaning was performed in Stata v17 (StataCorp, 2021) and analyses were performed in R version 4.2.2 (R Core Team, 2019), with the code publicly available at github.com/lwp501/RAPPORT.

#### Cross‐sectional impact of physical activity on psychological distress

Participants also reported physical activity at age 17. We consequently repeated the above analyses, estimating the average causal effect and variations in this effect (ATE and CATE) of meeting guidelines on physical activity at age 17 on concurrent psychological distress. We used the same set of confounders and methods as for the longitudinal analysis. The results from these analyses are available in Supporting Information S1: Section D.

## RESULTS

### Descriptive statistics

Descriptive statistics for the study analytic sample are shown in Table 1, with further details available in Section C: Table S2.

**TABLE 1:** Demographics of the sample, split by ‘exposure’ (self‐reported physical activity levels) at age 14.

	Total	Missing (%)	Didn't meet guideline at age 14	Met guideline at age 14
*N*	9123 (100%)	n/a	5722/9123 (62.7%)	3401/9123 (37.3%)
Male sex	4440/9123 (48.7%)	0/9123 (0%)	2400/5722 (41.9%)	2040/3401 (60.0%)
Ethnicity		72/9123 (0.79%)		
White	7166/9051 (79.2%)		4468/5681 (78.6%)	2698/3370 (80.1%)
Mixed	425/9051 (4.70%)		257/5681 (4.52%)	168/3370 (4.99%)
Indian	260/9051 (2.87%)		166/5681 (2.92%)	94/3370 (2.79%)
Pakistani and Bangladeshi	689/9051 (7.61%)		469/5681 (8.26%)	220/3370 (6.53%)
Black or Black British	294/9051 (3.25%)		193/5681 (3.40%)	101/3370 (3.00%)
Other ethnic group	217/9051 (2.40%)		128/5681 (2.25%)	89/3370 (2.64%)
Body mass index	21.41 (4.10) *n* = 8827	296/9123 (3.24%)	21.79 (4.34) *n* = 5503	20.78 (3.59) *n* = 3324
LGBTQ+ sexuality	969/9088 (10.7%)	35/9123 (0.38%)	751/5697 (13.2%)	218/3391 (6.43%)
Religion		3136/9123 (34.4%)		
None	3509/5987 (58.6%)		2279/3892 (58.6%)	1230/2095 (58.7%)
Christian	1825/5987 (30.5%)		1155/3892 (29.7%)	670/2095 (32.0%)
Muslim	495/5987 (8.27%)		350/3892 (8.99%)	145/2095 (6.92%)
Other	158/5987 (2.64%)		108/3892 (2.77%)	50/2095 (2.39%)
Kessler score at age 17	7.31 (4.91) *n* = 9123	n/a	7.71 (4.96) *n* = 5722	6.64 (4.76) *n* = 3401
Physical activity days per week		n/a		
Every day	1608/9123 (17.6%)			
5–6 days	1793/9123 (19.7%)			
3–4 days	3145/9123 (34.5%)			
1–2 days	2203/9123 (24.1%)			
Not at all	374/9123 (4.10%)			

Abbreviation: LGBTQ, Lesbian, Gay, Bisexual, Transgender, Queer or other.

Around one third of the sample met the pre‐defined threshold for physical activity levels (*n* = 3401, 37.3%) and were thus classed as ‘exposed’. Some differences were observed across exposure levels. For example, around 60% of those who reported the recommended levels of physical activity were male versus 42% of those who did not.

Those who met the guidelines at age 14 had an average K6 score of 6.64 at age 17, while those who did not meet the guidelines had an average K6 score of 7.71, an unadjusted difference of 1.08 points, a statistically significant difference (Mann–Whitney‐*U* testing, *p* < 0.001).

### Average treatment effect

The average effect of meeting government guidelines of physical activity at age 14 on psychological distress at age 17 (compared with not meeting the guidelines) was 0.11 points on the K6 scale (95% confidence interval [CI]: −0.09 to 0.31, *p* = 0.28). That is, at an overall population level, we observed no statistically significant effect of meeting physical activity guidelines at age 14 on psychological distress at age 17. Further technical details relating to these results are provided in Section C: Figures S2 and S3, Table S3.

### Sensitivity analysis over the exposure definition

**FIGURE 1:**
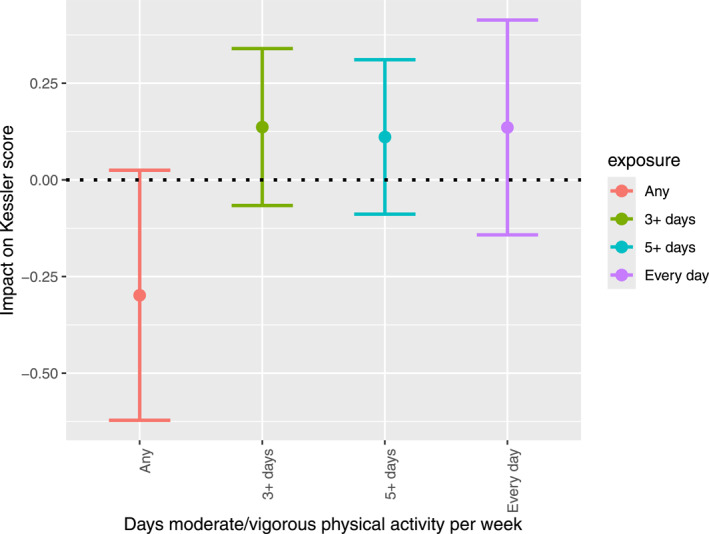
Estimates of the average ‘treatment’ effect of different definitions of ‘exposure’ to physical activity at age 14 on psychological distress at age 17.

Results from our sensitivity analysis, exploring the effect of varying cut‐offs for the definition of ‘recommended physical activity levels’ are shown in Figure 1. Full results are available in Section C: Table S4. As can be seen, no specific definition of ‘recommended physical activity levels’ was associated with a statistically significant impact on distress levels at age 17. However, there was a trend of borderline statistical significance when modelling the average effect of ‘any’ v ‘no’ self‐reported physical activity (average causal effect = −0.30, 95% CI −0.62 to 0.03, *p* = 0.07).

### Heterogeneity in the exposure effects

**FIGURE 2:**
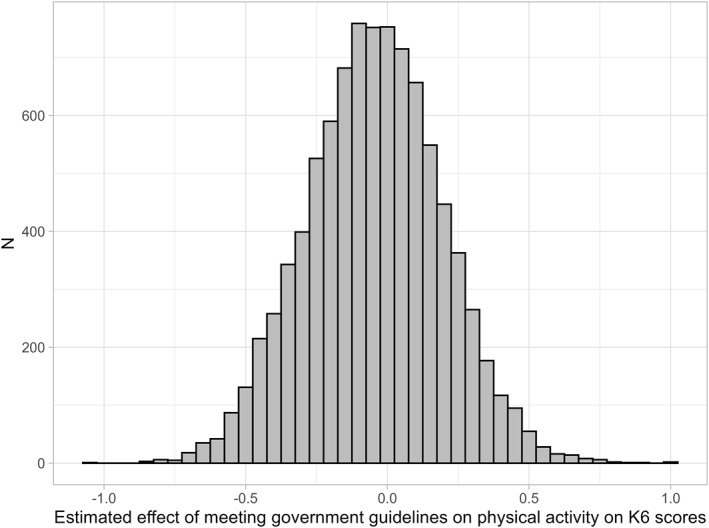
Distribution of the estimated individual treatment effects of physical activity at age 14 on psychological distress at age 17. K6, Kessler‐6 item.

Figure 2 displays the predicted treatment effects for the individuals in our sample from our causal forest model. Despite the average effect being close to zero, the estimated effect of physical activity levels on later psychological distress substantially varied.

Results from our multivariable linear regression model identified two statistically significant predictors of exposure‐effect heterogeneity. Males benefitted more, in terms of K6 score at 17, from meeting physical activity guidelines than females (*β* = −0.65, 95% CI −1.12 to −0.18, *p* = 0.01). That is, if we performed a subgroup analysis based on gender, we would expect the benefit from meeting government guidelines (vs. not meeting them) to differ between males and females, with males having lower K6 scores by approximately 0.65 points, holding all other covariates constant. Additionally, those with special educational needs benefited relatively less from meeting guidelines on physical activity than those without (*β* = 0.86, 0.15 to 1.58, *p* = 0.02). Those who attended a fee‐paying school borderline benefitted less than those who attended a state school (*β* = 0.78, −0.02 to 1.57, *p* = 0.05). Full results are available in Section C: Table S5.

### Cross‐sectional analysis results

The average effect of meeting guidelines for physical activity at age 17 on concurrent psychological distress was −0.39 (95% CI −0.61 to −0.18, *p* < 0.001). Thus, there was a modest, but statistically significant, cross‐sectional impact of meeting physical activity guidelines on K6 scores, compared to not meeting the guidelines. Sensitivity analyses suggested limited impact of changing the definition of ‘meeting guidelines’ on distress levels. There were no significant predictors of treatment heterogeneity, although body mass index (BMI) was of borderline statistical significance (*β* = −0.05, −0.11 to 0.00, *p* = 0.06). Full results are available in Section D: Tables S6–S9, Figures S4–S7.

## DISCUSSION

In this work we defined, in collaboration with a youth advisory group and lived experience experts, an a priori model exploring the relationship between physical activity at age 14 and psychological distress levels at 17. Using targeted learning, our results demonstrated no overall impact of meeting government guidelines on physical activity levels on psychological distress levels. Sensitivity analyses relating to the definition of the guidelines for physical activity did not identify a threshold at which there was a statistically significant average causal effect. Using causal forests, some significant variations in the effect of physical activity levels on subsequent psychological distress were noted. Specifically, males benefitted more from meeting government guidelines on physical activity, while those with special educational needs benefitted relatively less.

Our findings are somewhat in contrast to the majority of those reported by meta‐analytic studies of physical activity interventions on psychological outcomes, which report moderate to large effect sizes (Bailey et al., 2018; Biddle et al., 2019; Carter et al., 2016, 2021; Wang et al., 2022). However, this apparent disparity has a number of possible explanations. Effect sizes reported for physical activity‐based interventions on psychological outcomes are lower in general populations than in clinical populations (Carter et al., 2021; Mahindru et al., 2023). Indeed, there are numerous examples of well‐designed interventions that did not demonstrate modest, short‐term effects (Tymms et al., 2016). Secondly RCTs frequently deliver interventions in optimal circumstances (Zwarenstein & Treweek, 2009), and may only provide short‐term (weeks or a few months) follow‐up. Trials also generally evaluate the impact on psychological outcomes of a pre‐defined intervention of physical activity. This can be either as a single intervention or as a complex package of complementary interventions. In contrast, we evaluated the effect of natural exposure to physical activity, rather than short term adherence to an RCT protocol. It is possible the focussed and often complex nature of physical activity interventions delivered via RCTs have greater benefits, either direct and/or secondary (e.g., through enabling greater social contact between young people). As such, their results may not always translate to naturalistic settings where much of the activity may be informal, unplanned and possibly solitary, and therefore we may expect lower effect sizes in such settings.

We had data on self‐reported physical activity in the week before survey completion. From this, we classified whether an individual was likely meeting government guidelines relating to physical activity or not. It is feasible, as indicated by some RCT findings, that certain structured interventions aimed at increasing the amount of time spent doing moderate to vigorous physical activity improves psychological outcomes over the short to medium term in young people. However, it is equally plausible that, over a longer period the psychological benefits of physical activity do not endure. Adherence to long‐term health behavioural change, such as increasing physical activity, is difficult (Schwarzer, 2008). Evidence also suggests that engagement in physical activity declines over adolescence, before stabilising in young adulthood (van Sluijs et al., 2021). It is possible that the measure of physical activity analysed in this study is more representative of ‘typical’ long‐term physical activity levels in adolescents than those who are participating in an intervention in a trial, and thus a more plausible estimate of the potential longer‐term psychological impact of such activity.

Despite the lack of an overall, average population effect, we did identify some variations in the estimated impact of meeting guidelines on physical activity on later psychological distress. Our findings that males benefitted more than females is echoed in other, separate studies (Hale et al., 2021). It has been previously argued that gender may moderate the causal impact of physical activity on mental health outcomes. Specifically, it has been noted that studies reporting no effect used female‐only samples (Hale et al., 2021). Our findings also suggested that those individuals with special educational needs benefitted relatively less than those without, if they met the physical activity guidelines. It has previously been highlighted that factors such as barriers to inclusion and discrimination can inhibit enjoyment of physical education in those children with SEN (Coates & Vickerman, 2008). This may, to some extent at least, offer an explanation as to this finding.

A lived experience researcher has provided their interpretation of the results in Box 1.

**BOX 1 Interpretation of the results from a lived experience researcher.**

The finding of no overall effect reveals the complexity of poor mental health and its intersections. There are many contextual nuances around mental health, especially at the ages considered in this study, that could influence the self‐reported data, especially when collected generally and as a one‐off rather than across a period of time. I think these findings demonstrate that context and social determinants influence how certain groups of the population may benefit, or not, from general recommendations. This contrasts with the ‘one‐size‐fits‐all’ approaches often adopted when establishing governmental guidelines.

I do not find it surprising that young men may benefit more from physical activity. This is because the developmental stages at 14 and 17 years old differ considerably between men and women/people assigned female at birth. The latter often have an earlier start of adolescence and the hormonal changes that accompany it. Moreover, given that guidelines have often been designed by, and for, neurotypical populations, it is perhaps to be expected that neurodivergent and disabled individuals will not necessarily benefit from those strategies. Some may even experience counterproductive effects, due to the pressure to meet unrealistic expectations. Something that policy makers could take from these results is the relevance of having an intersectional, evidence‐based approach to tailoring public health recommendations. The policy development process should also integrate the views and recommendations of people with relevant lived experience.

The way this project involved lived‐experience researchers exemplifies the great potential of peer research to address systemic issues. Having the opportunity, as a lived experience researcher, to understand the proposed methodology and provide feedback to refine the ‘training of the machine’ and the DAG definitely seems like a good starting point to tackle the potential biases.

### Strengths and limitations

We used data drawn from a large, nationally representative cohort study, increasing the potential generalisability of our findings. We involved young people and those with relevant lived experience through co‐developing the DAG that described the theoretical model, in line with best practice guidance for developing causal models (Rodrigues et al., 2022). We drew on the expertise of our lived‐experience participants to interpret our findings. Our novel application of ‘doubly‐robust’ causal machine‐learning based methods provided an advantage over standard, regression‐based statistical methods.

The use of causal inference methods aimed to adjust for the lack of randomisation in the cohort study. However, as with all such approaches, the ability to make causal inferences from the results rests on the four assumptions listed in the methods section (Naimi & Whitcomb, 2023). We provide a reflection on these assumptions and whether we may meet them in this instance in Supporting Information S1: Section C. The key assumption of *conditional exchangeability* (‘no unmeasured confounding’) is not possible to test directly. However, our collaboration with the youth advisory group and lived‐experience experts defined an a priori model of the causal relationship between physical activity at the age of 14 and psychological distress at the age of 17. The extent of the data in the MCS allowed us to identify relevant covariates to include in our model. Nevertheless, it is still possible that not all the possible confounders were captured. Some caution must also be exercised in interpreting the results of our cross‐sectional analysis. This is particularly because the direction of causality cannot be firmly established. That is, those with lower levels of distress may have been more likely to choose to engage in more physical activity.

Even if our results reflect correlational rather than causal effects, they still offer insight into the potential impact of the exposure on the outcome of interest. Targeted learning has been shown to be more efficient than alternative modelling approaches, even in a correlational framework. This is because, unlike conventional statistical approaches, only the parameter of interest (the average causal effect, or ATE) needs to be estimated. Moreover, the method reduces the risk of model misspecification compared with conventional parametric modelling (Van der Laan & Rose, 2011). Thus, the results are less at risk of bias.

A limitation in the study is the reliance on self‐report data, including physical activity levels. Objective (e.g., GPS, or accelerometer) measures of physical activity have been previously compared to self‐report data in young people. It has been reported there is a tendency for adolescents to over‐report the amount of time spent in physical activity. Specifically, the average number of days where at least an hour of moderate to vigorous activity took place was recorded as 3.82 days per week via self‐report and only 2.34 days via accelerometer (Burchartz et al., 2021). Nevertheless, our findings should still stand if the bias is systematic, rather than random (i.e., if most participants tended to overestimate the amount of time spent in moderate to vigorous physical activity). Moreover, our sensitivity analysis demonstrated invariance to changing the definition of meeting the government guideline on physical activity, which would have accounted for a trend to over‐report. Nevertheless, a more objective measure of physical activity would have been more robust compared to self‐report alone. Additionally, more frequent data collections would have allowed for the shorter‐term effects of meeting government guidelines on physical activity to be estimated.

The majority of the data used were originally collected between 2015 and 2016. There have been many changing secular trends since then. For example, the change in the nature and time spent engaged in social media and other online activities (Odgers et al., 2020). Another minor weakness is we used a single, rather than multiple, imputation for missing data. It is currently unclear how to combine multiple imputation with targeted learning. However, missing values were not extensive in our analytic dataset, so this is unlikely to have significantly impacted on our findings. Additionally, to ensure interpretability, we did not include interaction terms in our linear regression model for exposure‐effect heterogeneity. We may therefore not have identified important statistically significant interaction terms for this heterogeneity. This could be addressed in future research by, for example, implementing a LASSO regression that selects relevant interactions among covariates. Finally, another potential limitation is that two of our confounders (sexuality and religion) were recorded at age 17. However, we assume these characteristics remained constant between ages 14 and 17. Similarly, some of our confounders (e.g., self‐reported hours spent on social networking sites) are measured concurrently with the exposure. Our work with our youth advisory group means we are confident that these variables are most appropriately conceptualised as confounders rather than mediators. Nevertheless, ideally these constructs would have been measured before the exposure.

### Implications for policy

Our findings that there are variations in the impact of physical activity on certain subgroups has implications for policy and practice. Initiatives aimed at improving mental health via increased physical activity could be targeted at those groups, such as males, who may benefit more. There was also some suggestion, from our cross‐sectional findings, that those young people who have higher than average BMIs, may especially benefit from increasing physical activity levels. This is especially relevant given the previously curvilinear relationship described between childhood BMI and mental health (Tiffin et al., 2011).

### Potential areas for future research

Further research could focus on replicating these findings in other datasets. This may include, where feasible, conducting sub‐analyses of data from relevant, previously conducted large scale trials. These could assess for evidence of particular benefit for the groups of adolescents identified in our study.

Intervention‐based research should continue to develop tailored approaches to supporting specific groups of young people to increase time spent in moderate to vigorous activity, both formally and informally. Some promising approaches have already been evaluated for feasibility, such as a Behavioural Activation based approach, targeting young people who are overweight or obese (Arnott et al., 2020). Advances in interactive and immersive technology also shows promise for engaging young people, who may have been less motivated by traditional PE, in physical activity (Baranowski et al., 2012).

Our use of two causal machine learning methods demonstrates the potential application of these methods to youth mental health research. In particular, causal forests could provide valuable insights into group differences in response to an exposure or intervention. In line with their applications in other fields, these methods offer the potential to rapidly derive personalised, policy‐relevant findings from observational youth mental health data. Future research should work to identify suitable data to exploit and validate these methods in this field. In addition to research‐derived observational data, there is an increasing availability of routinely‐arising data on youth mental health and wellbeing. These may be obtained from health, education and social care records, which may be linked. The use of causal machine learning methods to derive policy‐relevant insights would be far more rapid than a reliance on trial‐based research. The duration of a trial, from conception through to final reporting, is multiple years. By this time the findings may no longer be applicable. In contrast, causal machine learning methods could be deployed in near‐real time.

## CONCLUSION

We observed no overall long‐term impact of physical activity levels reported at age 14 on youth distress reported at age 17; however, there was a modest positive short‐term impact of physical activity levels on concurrent distress levels. Boys may particularly benefit from interventions supporting increased physical activity. On a cautionary note, we may need to tailor physical activity for young people with special education needs so it does not increase their distress. This is the first mental health study to apply causal machine learning based on a model co‐produced with lived‐experience experts. This is a novel and promising alternative to RCTs as a way of rapidly generating evidence from observational youth mental health data.

## AUTHOR CONTRIBUTIONS

**Lewis W. Paton**: Conceptualization; data curation; formal analysis; funding acquisition; methodology; software; visualization; writing—original draft; writing—review and editing. **Noemi Kreif**: Conceptualization; formal analysis; funding acquisition; methodology; supervision; writing—original draft; writing—review and editing. **Lauren M. E. Aylott**: Investigation; methodology; supervision; writing—original draft; writing—review and editing. **Philip Kerrigan**: Investigation; methodology; supervision; writing—original draft; writing—review and editing. **Clau Nader**: Investigation; methodology; writing—original draft; writing—review and editing. **Lina Gega**: Conceptualization; funding acquisition; supervision; writing—original draft; writing—review and editing. **Paul A. Tiffin**: Conceptualization; funding acquisition; methodology; project administration; supervision; writing—original draft; writing—review and editing.

## CONFLICT OF INTEREST STATEMENT

The authors declare no conflicts of interest.

## ETHICAL CONSIDERATIONS

This study was a secondary analysis of de‐identified data and exempt from ethical approval. This was confirmed in writing by the Chair of the University of York's Department of Health Sciences Research Governance Committee.

## Supporting information

Supporting Information S1

## Data Availability

Data used in this study are available from the UK Data Service (https://ukdataservice.ac.uk/). The code used to clean and analyse the data are available from GitHub (https://github.com/lwp501/RAPPORT).
