# Supplementary material for: The impact of physical activity on psychological distress in young people: An analysis of longitudinal data from the Millennium Cohort Study using causal machine learning
Source: JCPP Adv. 2025 Aug 19;6(2):e70035. doi: 10.1002/jcv2.70035 (PMC13260710; doi:10.1002/jcv2.70035)
Supplement: Supplementary file 1 — Supporting Information S1 [file JCV2-6-e70035-s001.docx]

**SUPPORTING INFORMATION: THE IMPACT OF PHYSICAL ACTIVITY ON PSYCHOLOGICAL DISTRESS IN YOUNG PEOPLE: AN ANALYSIS OF LONGITUDINAL DATA FROM THE MILLENNIUM COHORT STUDY USING CAUSAL MACHINE LEARNING**

Lewis W Paton, Noemi Kreif, Lauren M E Aylott, Philip Kerrigan, Clau Nader, Lina Gega and Paul A Tiffin.

**SUPPORTING INFORMATION, SECTION A: FULL DETAILS OF COVARIATES**

***Patient and Public Involvement***

To define the set of confounders to be used, we co-produced a directed acyclic graph (DAG) with a youth advisory group and individuals with relevant lived experience. A DAG is a graphical representation of the hypothetical causal relationship between variables and can help define the confounders needed to account for in the modelling process. Domain experts have been used previously during DAG development to help develop a more realistic representation of the theoretical model under study (Rodrigues, Kreif, Lawrence-Jones, Barahona, & Mayer, 2022). This proceeded in two stages.

We initially met with a Youth Opinion and Advisory Group, a group of young people who actively participate in research. Some have lived experience of mental illness. To identify potential confounders, we asked the group to consider what factors may influence whether a 14-year-old exercises, and whether these variables might also independently influence their mood. During this consultation, the group identified a range of potential confounders including those relating to physical health, psychological health and social connectedness.

Subsequently, four representatives - two young adults with lived experience of mental illness and two parents who were carers of young people with mental illness - joined the team as lay researchers and had input throughout the project. Recognising the need for lay researchers to understand the context and enough terminology to contribute to the discussion (Staley, 2013), we initially provided some training regarding confounders and DAGs. The lay researchers were asked to review the confounding factors identified via the Youth Opinion and Advisory Group.

This process culminated in identifying eight categories of potential confounders (Figure S1). We thus explored the list of variables collected at ages 14 and 17 in the Millennium Cohort Study (MCS) to identify those variables which belonged to these categories. Where possible, we included data from age 14 to ensure the correct temporal ordering between the confounders and the outcome. We identified 24 potential confounders for inclusion in our analyses (Table S1). These were a mixture of self & parent-proxy reported, as well as data relating to parental mental health, and home and school environments. Where possible we included all categories as originally collected. However, some categories needed to be collapsed to ensure sufficient numbers of observations in each category.


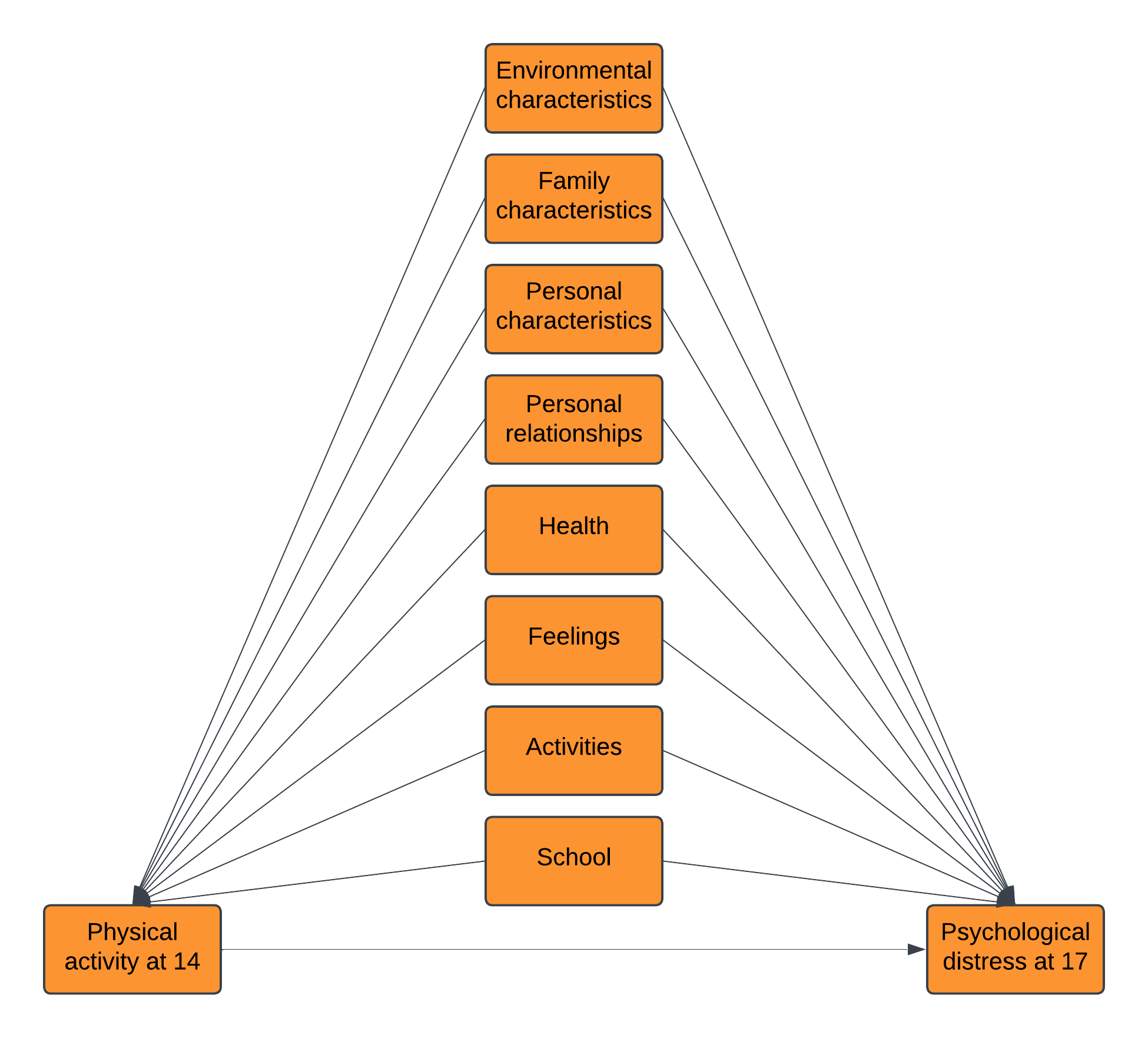


**Figure S1**: Directed Acyclic Graph of the relationship between physical activity and psychological distress, co-developed with our youth advisory and lived experience groups.

| **MCS Variable name** | **Variable** | **Confounder category** | **Data type** | **Details** |
| --- | --- | --- | --- | --- |
| FCCSEX00 | Sex | Personal characteristics | Binary | Self-reported. Male, female |
| FDCE0600 | Ethnicity | Personal characteristics | Nominal | Self-reported. 6 categories: white, mixed, Indian, Pakistani and Bangladeshi, Black/Black British, other ethnic group |
| GCSXID00 | Sexuality (age 17) | Personal characteristics | Binary | Self-reported. Only collected at age 17. Completely/mainly heterosexual, any other reported sexuality |
| GCRELE00_R20 | Religion (age 17) | Personal characteristics | Nominal | Self-reported. Only collected at age 17. Four categories: None, Christian, Muslim, other |
| FCBMIN6 | Body Mass Index | Personal characteristics | Continuous | kg/m2 |
| FCHARM00 | In the past year has CM self-harmed | Health | Binary | Self-reported. Yes, no |
| FPCLSI00 | if CM has longstanding illness | Health | Binary | Parent-reported. Yes, no |
| FCGDPE00 | I am good at PE | School | Binary | Answers to statement “I am good at physical education”: agree/strongly agree, Disagree/strongly disagree |
| FCSCWK00, FCWYLK00, FCFMLY00, FCFRNS00,  FCSCHL00, FCLIFE00 | Total life satisfaction score | Feelings | Continuous | Measured by six items each focusing on a different aspect of life, each with seven responses. Total score, ranging from six to 42 was used (Bannink, Pearce, & Hope, 2016) |
| FCMDSA00, FCMDSB00, FCMDSC00, FCMDSD00, FCMDSE00, FCMDSF00 FCMDSG00, FCMDSH00,  FCMDSI00, FCMDSJ00, FCMDSK00, FCMDSL00, FCMDSM00 | Short Moods and Feelings Questionnaire score | Health | Continuous | 13 items, each scored from 0 to 2. Total score, ranging from 0 to 26, was used (Angold, Costello, Messer, & Pickles, 1995) |
| FCSATI00, FCGDQL00, FCDOWL00, FCVALU00, FCGDSF00 | Rosenberg Self-Esteem scale score | Feelings | Continuous | Five items each on a four point scale. Total score, ranging from five to 20, was used. the Rosenberg Self-Esteem scale (Rosenberg, 1965) |
| FCNUFR00 | Do you have any close friends? | Personal relationships | Binary | Self-reported. Yes, No. |
| FCSAFD00 | Safe for CM to walk/play etc within a mile/20 min from home | Environmental characteristics | Binary | Self-reported. Safe/very safe, not very safe/not at all safe |
| FCSOME00 | Self-reported hours per week spent on social networking sites | Activities | Ordinal | Self-reported. Eight categories: none, less than half an hour, half an hour to less than 1 hour, 1 hour to less than 2 hours, 2 hours to less than 3 hours, 3 hours to less than 5 hours, 5 hours to less than 7 hours, 7 hours or more |
| FCTVHO00 | self-reported hours per day of TV usage | Activities | Ordinal | Self-reported. Seven categories: Less than half an hour, half an hour to less than 1 hour, 1 hour to less than 2 hours, 2 hours to less than 3 hours, 3 hours to less than 5 hours, 5 hours to less than 7 hours, 7 hours or more |
| FCCOMH00 | self-reported hours per day videogame usage | Activities | Ordinal | Self-reported. Eight categories: None, less than half an hour, half an hour to less than 1 hour, 1 hour to less than 2 hours, 2 hours to less than 3 hours, 3 hours to less than 5 hours, 5 hours to less than 7 hours, 7 hours or more |
| FCINTH00 | self-reported hours of internet usage per day at home | Activities | Ordinal, 8 categories | Self-reported. Seven categories: Less than half an hour, half an hour to less than 1 hour, 1 hour to less than 2 hours, 2 hours to less than 3 hours, 3 hours to less than 5 hours, 5 hours to less than 7 hours, 7 hours or more |
| FDKESSL | Parental Kessler score | Family characteristics | Continuous | Parental psychological distress, total score from zero to 24 |
| FIMDINCE | Income | Family characteristics | Ordinal | 2004 Index of Multiple Deprivation Income Domain Decile (Ministry of Housing Communites & Local Government, 2020) |
| FPCSEN00 | Has CMs school told you CM has special needs | School/Health | Binary | Paren-reported. Yes, no |
| FIERURUR | Urban classification | Environmental characteristics | Binary | Sparse, less sparse. Based on Office for National Statistics Rural Urban Classification (Office for National Statistics) |
| FPSCTY00 | Fee-paying school | School | Binary | Parent-reported. Yes, No. |
| FPSCSX00 | Single-sex school | School | Binary | Parent-reported. Yes, No. |
| FPFTHS00 | Faith school | School | Binary | Parent-reported. Yes, No. |

**Table S1:** Full details on the covariates included in our analysis.

**SUPPORTING INFORMATION, SECTION B: ADDITIONAL METHODOLOGICAL DETAILS**

Causal modelling methods rely on statistically adjusting for any confounding present. In a doubly robust method, we estimate two models. One model predicts the outcome from the exposure status (exposed/non-exposed) and the values of the covariates. The other model predicts exposure status from the values of the covariates. These models are combined in a way that means our estimate of the exposure effect is robust to misspecification to either one of these models (Funk, Westreich, Wiesen, Stürmer, Brookhart, & Davidian, 2011).

Causal modelling using observational data rests on four assumptions (Naimi & Whitcomb, 2023):

- *Counterfactual consistency*: the observed outcome corresponds to the potential outcome under the observed treatment/exposure. i.e. those that engage in higher exercise levels at 14 *will* be observed to have the counterfactual distress levels at 17 that corresponds to higher exercise levels. This assumption implies the exposure (high level of exercise) is well defined and there exists only one version of it.
- *No interference*: the potential outcome for a given individual does not depend on the exposure status of any other individual.
- *Conditional exchangeability*: no unmeasured confounding is present.
- *Positivity*: everyone in the population of interest has some chance to be both exposed and unexposed. This implies exposed and unexposed individuals are present within all confounder levels (i.e., the probability of an exposure status is non-zero for all variables).

Our analytic sample contained n=9,123 individuals with available Kessler scores at 17, and self-reported physical activity levels at the age of 14. Data cleaning was performed in Stata v17 (StataCorp, 2021) and analyses were performed in R version 4.2.2 (R Core Team, 2019), with code available at <https://github.com/lwp501/RAPPORT>.

***Estimation of the Average ‘Treatment’ Effect***

Targeted learning was used to estimate the ATE (Schuler & Rose, 2017), using the *tmle* function from the *TMLE* package in R (Gruber & Laan, 2012). We used machine learning to predict both the probability that a particular individual was ‘exposed’ (the ‘propensity score’) and also the outcome of interest (conditional on exposure and covariates), separately. The initial estimate of the predicted outcomes are then adjusted according to the propensity score. Finally, the differences between the adjusted predicted outcomes under treated and control states are averaged out over the dataset. This procedure aims to reduce bias in the estimated ATE, compared to using the unadjusted predictions. We used an ‘ensemble’ of machine learning algorithms for both these models in the targeted learning process. This approach maximises predictive performance by combining the predictions from multiple algorithms (Van der Laan, Polley, & Hubbard, 2007). The predictions are then recombined. We used the *superlearne*r function in the *tmle* package to implement our ensemble models. In the present case each ensemble consisted of:

- A generalised linear model
- Generalised additive models, of degrees 2, 3, 4 and 5
- A penalised regression model, and
- A boosted tree algorithm (XGBoost (Chen & Guestrin, 2016)).

The final model chosen is the weighted average of these seven models that maximises predictive performance. The optimal weight is identified using ‘cross-validation’. Cross-validation is a resampling approach whereby the data are divided into multiple parts or “folds”. The model is trained on some folds and tested on the remaining fold and repeated so each fold is used as a test set once.

***Estimation of heterogeneity in the treatment effect using Causal Forests***

We used causal forests (Wager & Athey, 2018) to estimate heterogeneity in the treatment effect (also referred to as the conditional average treatment effect- CATE). Causal forests (Wager & Athey, 2018) build a series of generalised decision trees. In contrast to decision trees, a causal tree attempts to split the data in a way that the partitions result in ‘leaves’ where treatment effect is homogenous. This, simultaneously, results in differences in average treatment effects between ‘leaves’. A causal forest is the average of many causal trees, which differ due to randomly sampling the variables to base the splitting on and the random subsample of data used in each tree. The use of averaging across many trees is a way of addressing the ‘bias variance trade-off’ (Breiman, 2001).

We estimated the CATE using causal forests, using the *grf* package in R (Tibshirani, Athey, Sverdruo, & Wager, 2022). The number of trees in each forest was set at 2000, and the other hyperparameters (model settings) were tuned using cross-validation.

To identify the predictors of heterogeneity in the treatment effect, a multivariable linear regression model was used. The dependent variable was a transformation of the fitted values from the causal forest, the so-called “doubly-robust score”. To aid interpretation of the results all ordinal independent variables were converted to binary variables (values above the median response being defined as 1, and lower values as zero). For nominal variables such as ethnicity and religion, this categorisation was according to the majority vs other classes.

**SUPPORTING INFORMATION, SECTION C: THE EFFECT OF PHYSICAL ACTIVITY AT AGE 14 ON PSYCHOLOGICAL DISTRESS AT AGE 17 - FULL DESCRIPTIVE STATISTICS AND ADDITIONAL RESULTS**

|  | **Overall**  **(n=9123)** | **Didn’t meet guideline**  **(n=5722)** | **Met guideline**  **(n=3401)** |
| --- | --- | --- | --- |
| **Sex (%)** |  |  |  |
| Male | 4440  (48.7) | 2400 (41.9) | 2040 (60.0) |
| Female | 4683  (51.3) | 3322 (58.1) | 1361 (40.0) |
| Missing | 0 (0) | 0 (0) | 0 (0) |
| **Ethnicity (%)** |  |  |  |
| White | 7166  (78.5) | 4468 (78.1) | 2698 (79.3) |
| Mixed | 425 (4.7) | 257 (4.5) | 168 (4.9) |
| Indian | 260 (2.8) | 166 (2.9) | 94 (2.8) |
| Pakistani & Bangladeshi | 689 (7.6) | 469 (8.2) | 220 (6.5) |
| Black or Black British | 294 (3.2) | 193 (3.4) | 101 (3.0) |
| Other ethnic group | 217 (2.4) | 128 (2.2) | 89 (2.6) |
| Missing | 72 (0.8) | 41 (0.7) | 31 (0.9) |
| **Sexuality (%)** |  |  |  |
| Completely/mostly heterosexual | 8119  (89.0) | 4946 (86.4) | 3173 (93.3) |
| Any other sexuality | 969 (10.6) | 751 (13.1) | 218 (6.4) |
| Missing | 35 (0.4) | 25 (0.4) | 10 (0.3) |
| **Religion (%)** |  |  | |
| None | 3509  (38.5) | 2279 (39.8) | 1230 (36.2) |
| Christian | 1825 (20.0) | 1155 (20.2) | 670 (19.7) |
| Muslim | 495 (5.4) | 350 (6.1) | 145 (4.3) |
| Other | 158 (1.7) | 108 (1.9) | 50 (1.5) |
| Missing | 3136 (34.4) | 1830 (32.0) | 1306 (38.4) |
| **Body Mass Index (mean (SD))** | 21.41 (4.10) | 21.79 (4.34) | 20.78 (3.59) |
| Missing (%) | 296 (3.24) | 219 (3.83) | 77 (2.26) |
| **In the past year, has CM self-harmed (%)** |  |  | |
| No | 7637 (83.7) | 4722 (82.5) | 2915 (85.7) |
| Yes | 1350 (14.8) | 913 (16.0) | 437 (12.8) |
| Missing | 136 (1.5) | 87 (1.5) | 49 (1.4) |
| **Self-reported hours per week of social media usage (%)** |  |  | |
| None | 734 (8.0) | 509 (8.9) | 225 (6.6) |
| Less than half hour | 1157 (12.7) | 711 (12.4) | 446 (13.1) |
| Half an hour to less than 1 hour | 1358 (14.9) | 814 (14.2) | 544 (16.0) |
| 1 hour to less than 2 hours | 1554 (17.0) | 964 (16.8) | 590 (17.3) |
| 2 hours to less than 3 hours | 1417 (15.5) | 872 (15.2) | 545 (16.0) |
| 3 hours to less than 5 hours | 1267 (13.9) | 792 (13.8) | 475 (14.0) |
| 5 hours to less than 7 hours | 850 (9.3) | 559 (9.8) | 291 (8.6) |
| 7 hours or more | 783 (8.6) | 500 (8.7) | 283 (8.3) |
| Missing | 3 (0.0) | 1 (0.0) | 2 (0.1) |
| **Self-reported hours per day of TV usage (%)** |  |  | |
| Less than half hour | 393 (4.3) | 196 (3.4) | 197 (5.8) |
| Half an hour to less than 1 hour | 790 (8.7) | 411 (7.2) | 379 (11.1) |
| 1 hour to less than 2 hours | 1844 (20.2) | 1061 (18.5) | 783 (23.0) |
| 2 hours to less than 3 hours | 2192 (24.0) | 1369 (23.9) | 823 (24.2) |
| 3 hours to less than 5 hours | 2165 (23.7) | 1479 (25.8) | 686 (20.2) |
| 5 hours to less than 7 hours | 990 (10.9) | 686 (12.0) | 304 (8.9) |
| 7 hours or more | 743 (8.1) | 519 (9.1) | 224 (6.6) |
| Missing | 6 (0.1) | 1 (0.0) | 5 (0.1) |
| **Self-reported hours per day videogame usage (%)** |  |  | |
| None | 1754 (19.2) | 1129 (19.7) | 625 (18.4) |
| Less than half hour | 1291 (14.2) | 827 (14.5) | 464 (13.6) |
| Half an hour to less than 1 hour | 1064 (11.7) | 659 (11.5) | 405 (11.9) |
| 1 hour to less than 2 hours | 1394 (15.3) | 790 (13.8) | 604 (17.8) |
| 2 hours to less than 3 hours | 1234 (13.5) | 750 (13.1) | 484 (14.2) |
| 3 hours to less than 5 hours | 1152 (12.6) | 729 (12.7) | 423 (12.4) |
| 5 hours to less than 7 hours | 600 (6.6) | 394 (6.9) | 206 (6.1) |
| 7 hours or more | 630 (6.9) | 443 (7.7) | 187 (5.5) |
| Missing | 4 (0.0) | 1 (0.0) | 3 (0.1) |
| **Self-reported hours of internet usage per day at home (%)** |  |  | |
| Less than half hour | 339 (3.7) | 184 (3.2) | 155 (4.6) |
| Half an hour to less than 1 hour | 682 (7.5) | 377 (6.6) | 305 (9.0) |
| 1 hour to less than 2 hours | 1343 (14.7) | 718 (12.5) | 625 (18.4) |
| 2 hours to less than 3 hours | 1708 (18.7) | 1081 (18.9) | 627 (18.4) |
| 3 hours to less than 5 hours | 1985 (21.8) | 1270 (22.2) | 715 (21.0) |
| 5 hours to less than 7 hours | 1443 (15.8) | 968 (16.9) | 475 (14.0) |
| 7 hours or more | 1621 (17.8) | 1124 (19.6) | 497 (14.6) |
| Missing | 2 (0.0) | 0 (0.0) | 2 (0.1) |
| **I am good at PE (%)** |  |  | |
| Disagree/strongly disagree | 2260 (24.8) | 1884 (32.9) | 376 (11.1) |
| Agree/strongly agree | 6853 (75.1) | 3833 (67.0) | 3020 (88.8) |
| Missing | 10 (0.1) | 5 (0.1) | 5 (0.1) |
| **Do you have any close friends? (%)** |  |  | |
| No | 269 (2.9) | 190 (3.3) | 79 (2.3) |
| Yes | 8835 (96.8) | 5526 (96.6) | 3309 (97.3) |
| Missing | 19 (0.2) | 6 (0.1) | 13 (0.4) |
| **Rosenberg Self-Esteem scale score (mean (SD))** | 15.59 (2.90) | 15.20 (2.90) | 16.24 (2.77) |
| Missing | 229 (2.51) | 146 (2.55) | 83 (2.44) |
| **Fee paying school (%)** |  |  | |
| No | 8393 (92.0) | 5324 (93.0) | 3069 (90.2) |
| Yes | 636 (7.0) | 334 (5.8) | 302 (8.9) |
| Missing | 94 (1.0) | 64 (1.1) | 30 (0.9) |
| **Single sex school (%)** |  |  | |
| No | 7907 (86.7) | 4938 (86.3) | 2969 (87.3) |
| Yes | 1122 (12.3) | 720 (12.6) | 402 (11.8) |
| Missing | 94 (1.0) | 64 (1.1) | 30 (0.9) |
| **Faith school (%)** |  |  | |
| No | 6920 (75.9) | 4368 (76.3) | 2552 (75.0) |
| Yes | 1266 (13.9) | 808 (14.1) | 458 (13.5) |
| Missing | 937 (10.3) | 546 (9.5) | 391 (11.5) |
| **Longstanding illness (%)** |  |  | |
| No | 7610 (83.4) | 4727 (82.6) | 2883 (84.8) |
| Yes | 1443 (15.8) | 946 (16.5) | 497 (14.6) |
| Missing | 70 (0.8) | 49 (0.9) | 21 (0.6) |
| **Parental Kessler score (mean (SD))** | 4.25 (4.12) | 4.38 (4.19) | 4.03 (4.01) |
| Missing | 564 (6.18) | 379 (6.62) | 185 (5.44) |
| **Income decile (%)** |  |  | |
| 1 | 1120 (12.3) | 743 (13.0) | 377 (11.1) |
| 2 | 987 (10.8) | 630 (11.0) | 357 (10.5) |
| 3 | 895 (9.8) | 617 (10.8) | 278 (8.2) |
| 4 | 873 (9.6) | 552 (9.6) | 321 (9.4) |
| 5 | 795 (8.7) | 514 (9.0) | 281 (8.3) |
| 6 | 833 (9.1) | 528 (9.2) | 305 (9.0) |
| 7 | 817 (9.0) | 518 (9.1) | 299 (8.8) |
| 8 | 802 (8.8) | 483 (8.4) | 319 (9.4) |
| 9 | 946 (10.4) | 554 (9.7) | 392 (11.5) |
| 10 | 1037 (11.4) | 572 (10.0) | 465 (13.7) |
| Missing | 18 (0.2) | 11 (0.2) | 7 (0.2) |
| **Urban classification (%)** |  |  | |
| Sparse | 1507 (16.5) | 881 (15.4) | 626 (18.4) |
| Less sparse | 7598 (83.3) | 4830 (84.4) | 2768 (81.4) |
| NA | 18 (0.2) | 11 (0.2) | 7 (0.2) |
| **Short Moods and Feelings Questionnaire score (mean (SD))** | 5.57 (5.86) | 6.07 (6.04) | 4.73 (5.45) |
| Missing | 202 (2.21) | 129 (2.25) | 73 (2.15) |
| **Total life satisfaction score (mean (SD))** | 33.08 (6.58) | 32.50 (6.66) | 34.06 (6.32) |
| Missing | 168 (1.84) | 105 (1.84) | 63 (1.85) |
| **Safe for CM to walk/play etc within a mile/20 min from home (%)** |  |  | |
| Not very safe/not at all safe | 681 (7.5) | 447 (7.8) | 234 (6.9) |
| Safe/very safe | 8432 (92.4) | 5265 (92.0) | 3167 (93.1) |
| Missing | 10 (0.1) | 10 (0.2) | 0 (0.0) |
| **Has CMs school told you CM has special needs (%)** |  |  | |
| No | 8249 (90.4) | 5155 (90.1) | 3094 (91.0) |
| Yes | 780 (8.5) | 503 (8.8) | 277 (8.1) |
| Missing | 94 (1.0) | 64 (1.1) | 30 (0.9) |
| **Kessler score (age 17) (mean (SD))** | 7.31 (4.91) | 7.71 (4.96) | 6.64 (4.76) |

**Table S2:** Full descriptive statistics for the analytic sample, both overall and split by exposure status.

**
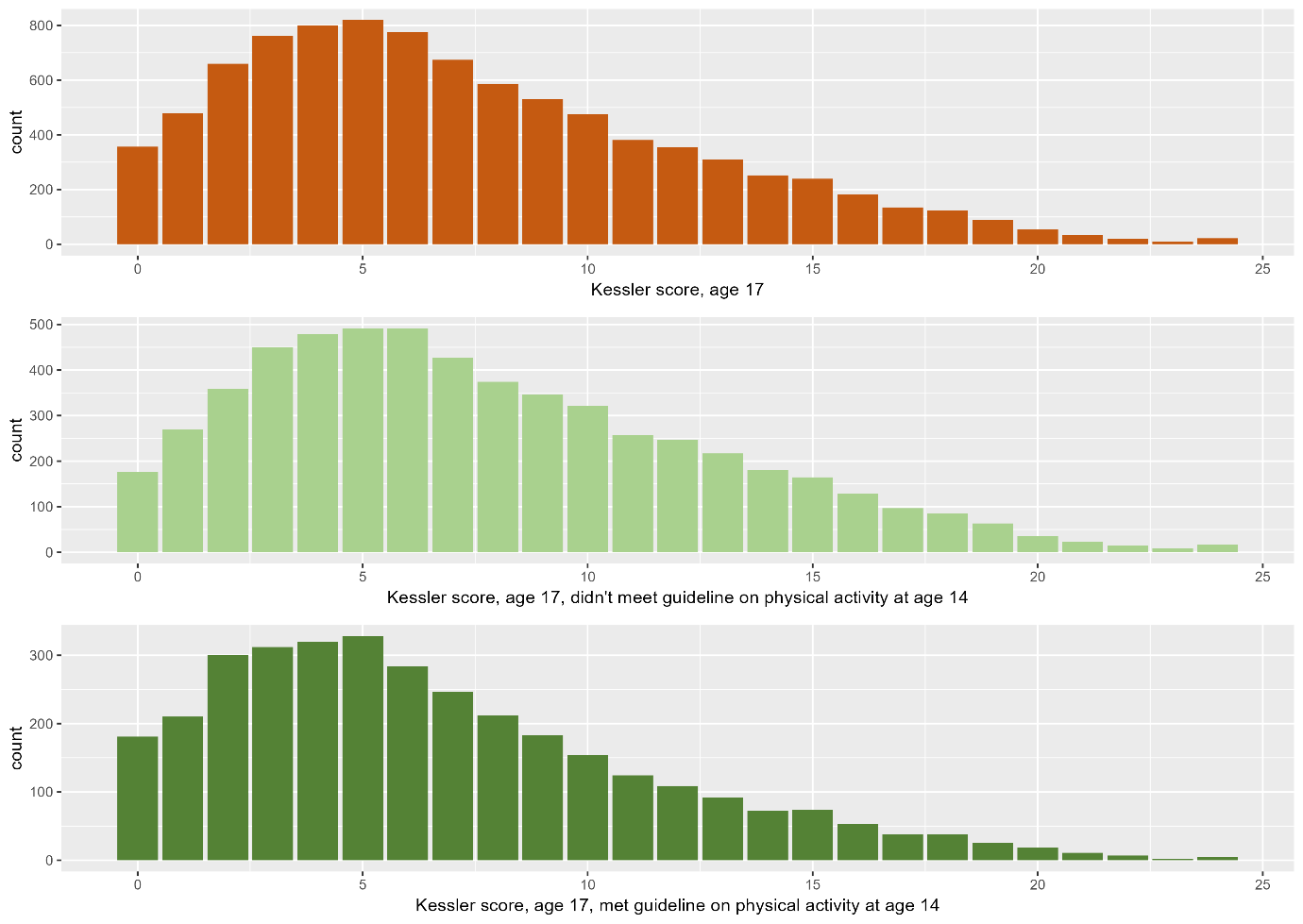
**

**Figure S2**: Distribution of Kessler Psychological Distress Scale scores at age 17 of the analytic sample.

***Propensity score distributions***


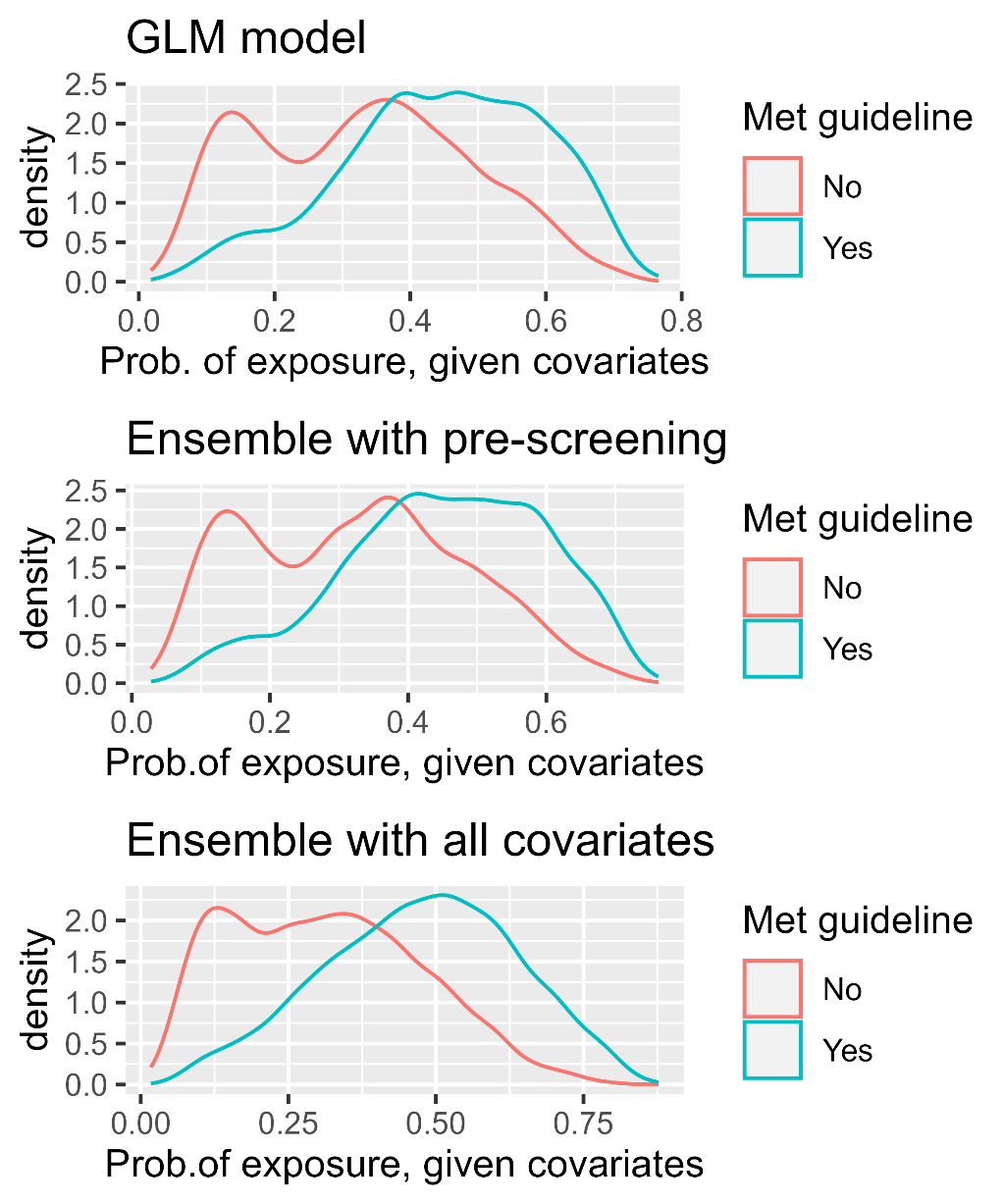


**Figure S3:** Propensity score distributions for each of the three targeted learning models: i) generalised linear model (GLM) only, ii) ensemble with pre-screening of covariates, iii) ensemble without pre-screening of covariates.

***Targeted learning ensemble weights***

|  | **Outcome** | **Exposure** |
| --- | --- | --- |
| **GAM2** | 0 | 0 |
| **GAM3** | 0 | 0 |
| **GAM4** | 0.48 | 0.14 |
| **GAM5** | 0 | 0.47 |
| **GLM** | 0 | 0 |
| **GLMNET** | 0.43 | 0.35 |
| **XGBOOST** | 0.09 | 0.04 |

**Table S3:** Ensemble weights in the targeted learning ensembles. GAM’X’ = generalised additive model of order ‘X’. GLM = generalised linear model, GLMNET = lasso and elastic net regularised model (Friedman et al., 2020).

***Results from alternative TMLE models***

- Generalised linear model only: ATE = 0.12, 95% confidence interval = -0.09 to 0.33, p=0.28.
- Ensemble with no pre-screening of covariates: ATE = 0.12, 95% confidence interval = -0.07 to 0.31, p=0.23.

***Sensitivity analysis for exposure definition***

| **Exposure definition** | **n (%) ‘exposed’** | **Average Treatment Effect** | **95% confidence interval** | **p-value** |
| --- | --- | --- | --- | --- |
| **Any (v none)** | 8749/9123 (95.9%) | -0.30 | -0.62 to 0.03 | 0.07 |
| **3 + days (v less than 3 days)** | 6546/9123 (71.8%) | 0.14 | -0.07 to 0.34 | 0.19 |
| **5+ days (primary result)** | 3401/9123 (37.3%) | 0.11 | -0.09 to 0.31 | 0.28 |
| **Every day** | 1608/9123 (17.6%) | 0.14 | -0.14 to 0.41 | 0.34 |

**Table S4:** Full results from sensitivity analysis over the definition of ‘exposed’.

*Causal forest tuned parameters*

- sample.fraction: 0.439355526061263
- mtry: 4
- min.node.size: 1
- honesty.fraction: 0.789559714030474
- honesty.prune.leaves: 1
- alpha: 0.0395969297387637
- imbalance.penalty: 0.266747472263799

***Linear predictors of heterogeneous treatment effects***

| **Variable** | **β** | **95% confidence interval** | **p-value** |
| --- | --- | --- | --- |
| Male gender | -0.65 | -1.12 to -0.18 | 0.01 |
| LGTBQ+ sexuality | 0.06 | -0.59 to 0.71 | 0.85 |
| Body Mass Index | 0.01 | -0.04 to 0.06 | 0.61 |
| In the past year CM has self-harmed | 0.18 | -0.46 to 0.81 | 0.58 |
| Above median self-reported hours per week of social media usage | -0.05 | -0.49 to 0.38 | 0.81 |
| Above median self-reported hours per day of TV | -0.25 | -0.67 to 0.18 | 0.26 |
| Above median self-reported hours per day of video games | 0.34 | -0.12 to 0.80 | 0.14 |
| Above median self-reported hours per day of internet usage | 0.41 | -0.06 to 0.88 | 0.09 |
| Agree/strongly agree ‘good at PE’ | -0.15 | -0.61 to 0.32 | 0.54 |
| Do you have any close friends? | -0.64 | -1.80 to 0.51 | 0.27 |
| Rosenberg Self-Esteem scale score | 0.06 | -0.03 to 0.15 | 0.18 |
| Fee-paying school | 0.78 | -0.02 to 1.57 | 0.05 |
| Single-sex school | -0.20 | -0.81 to 0.41 | 0.52 |
| Faith school | -0.24 | -0.78 to 0.29 | 0.37 |
| if CM has longstanding illness | -0.49 | -1.05 to 0.06 | 0.08 |
| Parental Kessler score | -0.03 | -0.08 to 0.02 | 0.19 |
| Above median Income | -0.2 | -0.62 to 0.21 | 0.34 |
| Urban classification | 0.09 | -0.45 to 0.63 | 0.75 |
| Short Moods and Feelings Questionnaire score | -0.01 | -0.05 to 0.04 | 0.79 |
| Total life satisfaction score | -0.02 | -0.06 to 0.02 | 0.42 |
| Safe for CM to walk/play etc within a mile/20 min from home | -0.63 | -1.38 to 0.11 | 0.10 |
| Has CMs school told you CM has special educational needs | 0.86 | 0.15 to 1.58 | 0.02 |
| Any religion | -0.36 | -0.79 to 0.06 | 0.09 |
| Non-white ethnicity | 0.37 | -0.17 to 0.90 | 0.18 |

**Table S5:** Full results from multivariable linear regression models for treatment effect heterogeneity of physical exercise at age 14 on psychological distress at age 17.

***Additional discussion - causal assumptions***

Causal modelling using observational data relies on four key assumptions – *counterfactual consistency*, *no interference*, *conditional exchangeability*, and *positivity.* Not all of these assumptions are readily testable. For example, that of *counterfactual consistency* can only be argued. However, it is still worth reflecting on any evidence, where available, to support or refute the other assumptions underlying causal inference. In terms of *positivity*, this is routinely evaluated as part of the targeted learning process. It can be seen in the descriptive statistics and the technical details provided in the Supporting Information that this assumption was supported. That is, all levels of confounding variables were observed for both ‘exposed’ and ‘non-exposed’ individuals.

Regarding the *no interference* assumption (the outcome for any individual does not depend on the exposure status of another individual): the extent to which physical activity may have an indirect impact on others is unclear. Whilst this effect cannot be ruled out, we attempted to assure the independence of observations by randomly selecting only one child from each family, where siblings were included in the MCS data.

The key assumption of *conditional exchangeability* (‘no unmeasured confounding’) is not possible to test directly. However, our collaboration with the youth advisory group and lived-experience experts defined an *a priori* model of the causal relationship between physical activity at the age of 14 with psychological distress at the age of 17. The extent of the data in the MCS allowed us to identify covariates to include in this model. Nevertheless, it is still possible that not all the possible confounders were captured. However, our main finding was of no longitudinal causal effect, with no suggestion of a trend. Thus, in order to overturn this result one or more unmeasured confounders, with very substantial single or combined relationships with both the outcome of interest and the exposure (physical activity levels), would need to have been omitted. Nevertheless, some caution must be exercised in interpreting the results of our cross-sectional analysis. The model used here was the same as for the longitudinal analysis. However, the role of the variables (e.g. moderators, mediators) may have differed. Moreover, in the cross-sectional analysis the direction of causality cannot be firmly established. That is, those with lower levels of distress may have been more likely to choose to engage in more physical activity.

**SUPPORTING INFORMATION, SECTION D: THE EFFECT OF PHYSICAL ACTIVITY AT AGE 17 ON PSYCHOLOGICAL DISTRESS AT AGE 17 - RESULTS**

***Demographic information***

|  | **Overall**  **(n=9090)** | **Didn’t meet guideline**  **(n=7166)** | **Met guideline**  **(n=1924)** |
| --- | --- | --- | --- |
| **Sex (%)** |  |  |  |
| Male | 4435 (48.8) | 3211 (44.8) | 1224 (63.6) |
| Female | 4655 (51.2) | 3955 (55.2) | 700 (36.4) |
| Missing | 0 (0) | 0 (0) | 0 (0) |
| **Ethnicity (%)** |  |  |  |
| White | 7060 (77.7) | 5507 (76.8) | 1553 (80.7) |
| Mixed | 424 (4.7) | 339 (4.7) | 85 (4.4) |
| Indian | 257 (2.8) | 209 (2.9) | 48 (2.5) |
| Pakistani & Bangladeshi | 684 (7.5) | 579 (8.1) | 105 (5.5) |
| Black or Black British | 289 (3.2) | 233 (3.3) | 56 (2.9) |
| Other ethnic group | 214 (2.4) | 170 (2.4) | 44 (2.3) |
| Missing | 162 (1.8) | 129 (1.8) | 33 (1.7) |
| **Sexuality (%)** |  |  |  |
| Completely/mostly heterosexual | 8088 (89.0) | 6316 (88.1) | 1772 (92.1) |
| Any other sexuality | 969 (10.7) | 823 (11.5) | 146 (7.6) |
| Missing | 33 (0.4) | 27 (0.4) | 6 (0.3) |
| **Religion (%)** |  |  | |
| None | 3539 (38.9) | 2807 (39.2) | 732 (38.0) |
| Christian | 1840 (20.2) | 1433 (20.0) | 407 (21.2) |
| Muslim | 501 (5.5) | 429 (6.0) | 72 (3.7) |
| Other | 158 (1.7) | 134 (1.9) | 24 (1.2) |
| Missing | 3052 (33.6) | 2363 (33.0) | 689 (35.8) |
| **Body Mass Index (mean (SD))** | 21.40 (4.09) | 21.49 (4.17) | 21.07 (3.79) |
| Missing (%) | 362 (3.9) | 299 (4.2) | 63 (3.3) |
| **In the past year, has CM self-harmed (%)** |  |  | |
| No | 7541 (83.0) | 5892 (82.2) | 1649 (85.7) |
| Yes | 1325 (14.6) | 1095 (15.3) | 230 (12.0) |
| Missing | 224 (2.5) | 179 (2.5) | 45 (2.3) |
| **Self-reported hours per week of social media usage (%)** |  |  | |
| None | 728 (8.0) | 574 (8.0) | 154 (8.0) |
| Less than half hour | 1147 (12.6) | 876 (12.2) | 271 (14.1) |
| Half an hour to less than 1 hour | 1347 (14.8) | 1045 (14.6) | 302 (15.7) |
| 1 hour to less than 2 hours | 1540 (16.9) | 1197 (16.7) | 343 (17.8) |
| 2 hours to less than 3 hours | 1387 (15.3) | 1094 (15.3) | 293 (15.2) |
| 3 hours to less than 5 hours | 1245 (13.7) | 970 (13.5) | 275 (14.3) |
| 5 hours to less than 7 hours | 838 (9.2) | 705 (9.8) | 133 (6.9) |
| 7 hours or more | 767 (8.4) | 629 (8.8) | 138 (7.2) |
| Missing | 91 (1.0) | 76 (1.1) | 15 (0.8) |
| **Self-reported hours per day of TV usage (%)** |  |  | |
| Less than half hour | 389 (4.3) | 271 (3.8) | 118 (6.1) |
| Half an hour to less than 1 hour | 783 (8.6) | 578 (8.1) | 205 (10.7) |
| 1 hour to less than 2 hours | 1825 (20.1) | 1394 (19.5) | 431 (22.4) |
| 2 hours to less than 3 hours | 2161 (23.8) | 1723 (24.0) | 438 (22.8) |
| 3 hours to less than 5 hours | 2141 (23.6) | 1723 (24.0) | 418 (21.7) |
| 5 hours to less than 7 hours | 966 (10.6) | 798 (11.1) | 168 (8.7) |
| 7 hours or more | 731 (8.0) | 602 (8.4) | 129 (6.7) |
| Missing | 94 (1.0) | 77 (1.1) | 17 (0.9) |
| **Self-reported hours per day videogame usage (%)** |  |  | |
| None | 1732 (19.1) | 1434 (20.0) | 298 (15.5) |
| Less than half hour | 1266 (13.9) | 1017 (14.2) | 249 (12.9) |
| Half an hour to less than 1 hour | 1054 (11.6) | 822 (11.5) | 232 (12.1) |
| 1 hour to less than 2 hours | 1376 (15.1) | 1052 (14.7) | 324 (16.8) |
| 2 hours to less than 3 hours | 1217 (13.4) | 937 (13.1) | 280 (14.6) |
| 3 hours to less than 5 hours | 1140 (12.5) | 885 (12.3) | 255 (13.3) |
| 5 hours to less than 7 hours | 592 (6.5) | 462 (6.4) | 130 (6.8) |
| 7 hours or more | 621 (6.8) | 480 (6.7) | 141 (7.3) |
| Missing | 92 (1.0) | 77 (1.1) | 15 (0.8) |
| **Self-reported hours of internet usage per day at home (%)** |  |  | |
| Less than half hour | 339 (3.7) | 239 (3.3) | 100 (5.2) |
| Half an hour to less than 1 hour | 675 (7.4) | 500 (7.0) | 175 (9.1) |
| 1 hour to less than 2 hours | 1328 (14.6) | 983 (13.7) | 345 (17.9) |
| 2 hours to less than 3 hours | 1690 (18.6) | 1325 (18.5) | 365 (19.0) |
| 3 hours to less than 5 hours | 1957 (21.5) | 1579 (22.0) | 378 (19.6) |
| 5 hours to less than 7 hours | 1422 (15.6) | 1152 (16.1) | 270 (14.0) |
| 7 hours or more | 1589 (17.5) | 1313 (18.3) | 276 (14.3) |
| Missing | 90 (1.0) | 75 (1.0) | 15 (0.8) |
| **I am good at PE (%)** |  |  | |
| Disagree/strongly disagree | 2220 (24.4) | 1908 (26.6) | 312 (16.2) |
| Agree/strongly agree | 6770 (74.5) | 5176 (72.2) | 1594 (82.8) |
| Missing | 100 (1.1) | 82 (1.1) | 18 (0.9) |
| **Do you have any close friends? (%)** |  |  | |
| No | 263 (2.9) | 214 (3.0) | 49 (2.5) |
| Yes | 8719 (95.9) | 6864 (95.8) | 1855 (96.4) |
| Missing | 108 (1.2) | 88 (1.2) | 20 (1.0) |
| **Rosenberg Self-Esteem scale score (mean (SD))** | 15.59 (2.90) | 15.43 (2.87) | 16.16 (2.93) |
| Missing | 316 (3.5) | 256 (3.6) | 60 (3.1) |
| **Fee paying school (%)** |  |  | |
| No | 8361 (92.0) | 6620 (92.4) | 1741 (90.5) |
| Yes | 631 (6.9) | 466 (6.5) | 165 (8.6) |
| Missing | 98 (1.1) | 80 (1.1) | 18 (0.9) |
| **Single sex school (%)** |  |  | |
| No | 7871 (86.6) | 6169 (86.1) | 1702 (88.5) |
| Yes | 1121 (12.3) | 917 (12.8) | 204 (10.6) |
| Missing | 98 (1.1) | 80 (1.1) | 18 (0.9) |
| **Faith school (%)** |  |  | |
| No | 6895 (75.9) | 5429 (75.8) | 1466 (76.2) |
| Yes | 1255 (13.8) | 976 (13.6) | 279 (14.5) |
| Missing | 940 (10.3) | 761 (10.6) | 179 (9.3) |
| **Longstanding illness (%)** |  |  | |
| No | 7567 (83.2) | 5955 (83.1) | 1612 (83.8) |
| Yes | 1448 (15.9) | 1147 (16.0) | 301 (15.6) |
| Missing | 75 (0.8) | 64 (0.9) | 11 (0.6) |
| **Parental Kessler score (mean (SD))** | 4.26 (4.13) | 4.28 (4.14) | 4.18 (4.06) |
| Missing | 572 (6.3) | 477 (6.7) | 95 (4.9) |
| **Income decile (%)** |  |  | |
| 1 | 1118 (12.3) | 901 (12.6) | 217 (11.3) |
| 2 | 982 (10.8) | 800 (11.2) | 182 (9.5) |
| 3 | 888 (9.8) | 715 (10.0) | 173 (9.0) |
| 4 | 865 (9.5) | 690 (9.6) | 175 (9.1) |
| 5 | 787 (8.7) | 605 (8.4) | 182 (9.5) |
| 6 | 836 (9.2) | 658 (9.2) | 178 (9.3) |
| 7 | 819 (9.0) | 657 (9.2) | 162 (8.4) |
| 8 | 801 (8.8) | 608 (8.5) | 193 (10.0) |
| 9 | 943 (10.4) | 736 (10.3) | 207 (10.8) |
| 10 | 1032 (11.4) | 783 (10.9) | 249 (12.9) |
| Missing | 19 (0.2) | 13 (0.2) | 6 (0.3) |
| **Urban classification (%)** |  |  | |
| Sparse | 1499 (16.5) | 1181 (16.5) | 318 (16.5) |
| Less sparse | 7572 (83.3) | 5972 (83.3) | 1600 (83.2) |
| NA | 19 (0.2) | 13 (0.2) | 6 (0.3) |
| **Short Moods and Feelings Questionnaire score (mean (SD))** | 5.57 (5.86) | 5.79 (5.94) | 4.74 (5.46) |
| Missing | 288 (3.2) | 229 (3.2) | 59 (3.1) |
| **Total life satisfaction score (mean (SD))** | 33.09 (6.58) | 32.85 (6.60) | 33.99 (6.46) |
| Missing | 255 (2.8) | 196 (2.7) | 59 (3.1) |
| **Safe for CM to walk/play etc within a mile/20 min from home (%)** |  |  | |
| Not very safe/not at all safe | 669 (7.4) | 525 (7.3) | 144 (7.5) |
| Safe/very safe | 8323 (91.6) | 6558 (91.5) | 1765 (91.7) |
| Missing | 98 (1.1) | 83 (1.2) | 15 (0.8) |
| **Has CMs school told you CM has special needs (%)** |  |  | |
| No | 8205 (90.3) | 6474 (90.3) | 1731 (90.0) |
| Yes | 787 (8.7) | 612 (8.5) | 175 (9.1) |
| Missing | 98 (1.1) | 80 (1.1) | 18 (0.9) |
| **Kessler score (age 17) (mean (SD))** | 7.27 (4.90) | 7.52 (4.93) | 6.35 (4.65) |

**Table S6:** Full descriptive statistics for the analytic sample for the cross-sectional analysis at age 17, both overall and split by exposure status.

***
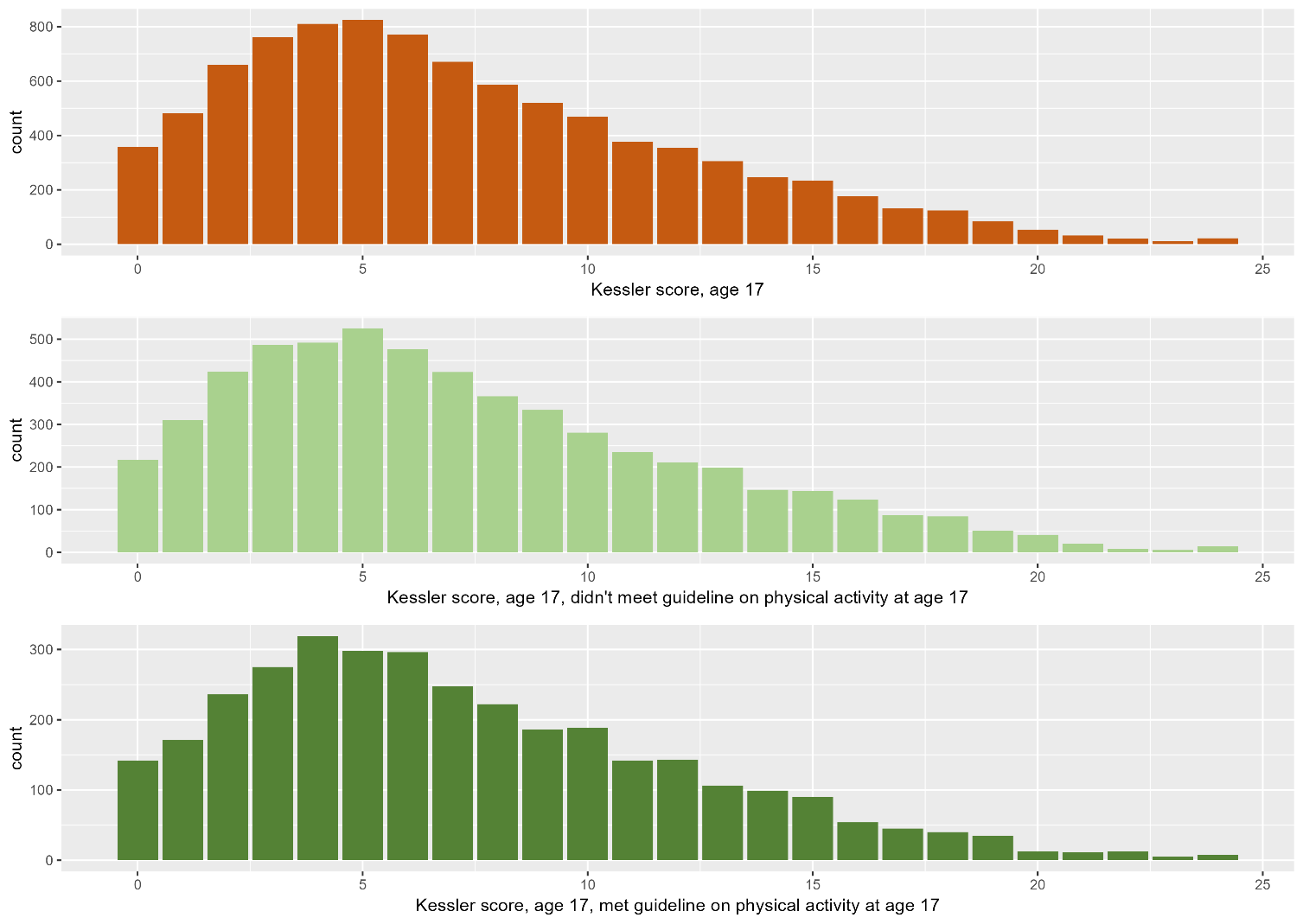
***

**Figure S4:** Distribution of Kessler Psychological Distress Scale scores at age 17 of the analytic sample.

***Propensity score distributions***


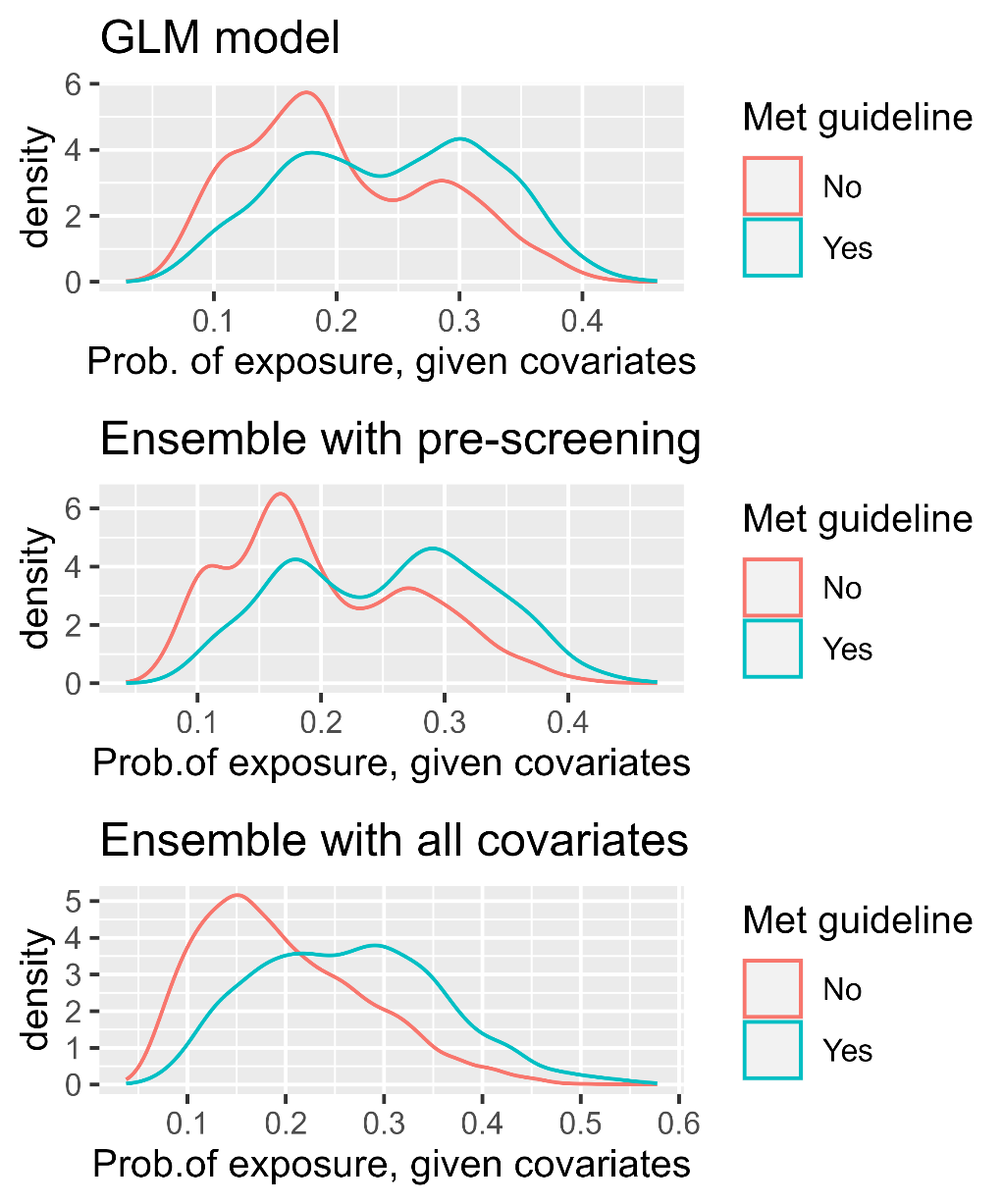


**Figure S5:** Propensity score distributions for each of the three targeted learning models: i) generalised linear model (GLM) only, ii) ensemble with pre-screening of covariates, iii) ensemble without pre-screening of covariates.

***Results from alternative targeted learning models***

- Generalised linear model only: ATE = -0.40, 95% confidence interval = -0.63 to -0.17, p<0.001.
- Ensemble with no pre-screening of covariates: ATE = -0.38, 95% confidence interval = -0.59 to -0.17, p=<0.001.

***Targeted learning ensemble weights***

|  | **Outcome** | **Exposure** |
| --- | --- | --- |
| **GAM2** | 0 | 0.58 |
| **GAM3** | 0.35 | 0.01 |
| **GAM4** | 0.24 | 0 |
| **GAM5** | 0 | 0 |
| **GLM** | 0 | 0 |
| **GLMNET** | 0.37 | 0.34 |
| **XGBOOST** | 0.05 | 0.07 |

**Table S7:** Ensemble weights in the targeted learning ensembles. GAM’X’ = generalised additive model of order ‘X’. GLM = generalised linear model, GLMNET = lasso and elastic net regularised model (Friedman et al., 2020).

***Full results from sensitivity analysis for exposure definition***

*
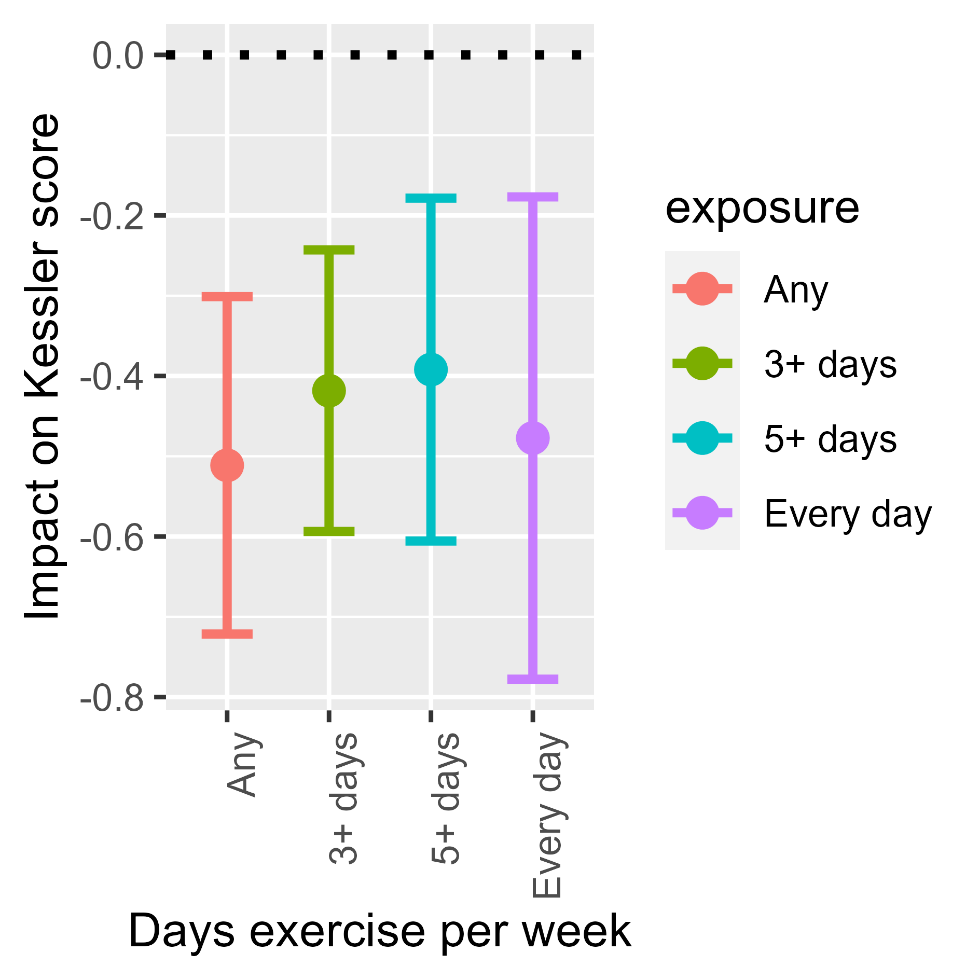
*

**Figure S6**: Estimates of the Average ‘Treatment’ Effect of different definitions of ‘exposure’ to physical activity at age 17 on mental distress at age 17.

| **Exposure definition** | **n (%) ‘exposed’** | **Average Treatment Effect** | **95% confidence interval** | **p-value** |
| --- | --- | --- | --- | --- |
| **Any (v none)** | 6894/9090 (75.8%) | -0.51 | -0.72 to -0.30 | p<0.001 |
| **3 + days (v less than 3 days)** | 4199/9090 (46.2%) | -0.42 | -0.59 to -0.24 | p<0.001 |
| **5+ days (primary result)** | 1924/9090 (21.2%) | -0.39 | -0.61 to -0.18 | p<0.001 |
| **Every day** | 889/9090 (9.78%) | -0.48 | -0.78 to -0.18 | p=0.002 |

**Table S8:** Full results from sensitivity analysis over the definition of ‘exposed’.

***Causal forest tuned parameters***

- sample.fraction: 0.355454131984152
- mtry: 28
- min.node.size: 1
- honesty.fraction: 0.500265690404922
- honesty.prune.leaves: 0
- alpha: 0.0152906331932172
- imbalance.penalty: 2.01928675338643

***Heterogeneity in treatment effect***

*
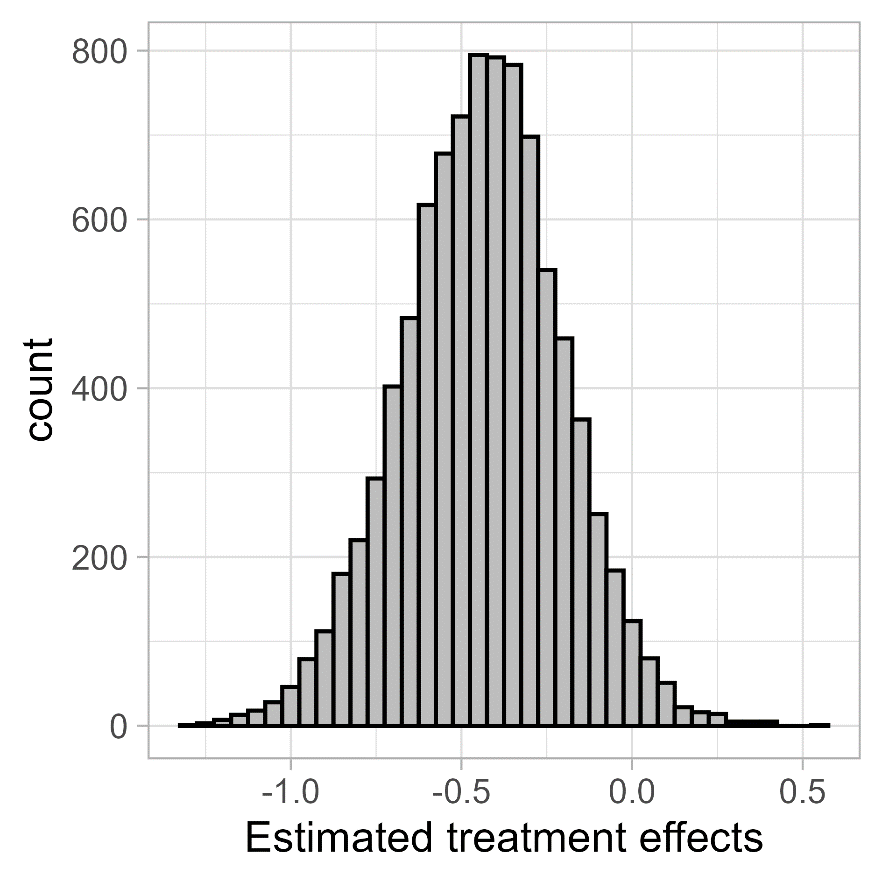
*

**Figure S7:** Distribution of the estimated individual treatment effects of physical exercise at age 17 on psychological distress at age 17

***Linear predictors of heterogeneous treatment effects***

| **Variable** | **β** | **95% confidence interval** | **p-value** |
| --- | --- | --- | --- |
| Male gender | -0.16 | -0.69 to 0.37 | 0.55 |
| LGTBQ+ sexuality | 0.59 | -0.14 to 1.33 | 0.11 |
| Body Mass Index | -0.05 | -0.11 to 0.00 | 0.06 |
| In the past year CM has self-harmed | 0.05 | -0.66 to 0.77 | 0.89 |
| Above median self-reported hours per week of social media usage | 0.15 | -0.34 to 0.64 | 0.56 |
| Above median self-reported hours per day of TV | -0.09 | -0.57 to 0.38 | 0.71 |
| Above median self-reported hours per day of video games | 0.42 | -0.10 to 0.94 | 0.11 |
| Above median self-reported hours per day of internet usage | -0.32 | -0.84 to 0.21 | 0.24 |
| Agree/strongly agree ‘good at PE’ | -0.16 | -0.68 to 0.37 | 0.56 |
| Do you have any close friends? | -0.38 | -1.67 to 0.92 | 0.57 |
| Rosenberg Self-Esteem scale score | 0.00 | -0.10 to 0.10 | 0.98 |
| Fee-paying school | -0.07 | -0.97 to 0.82 | 0.87 |
| Single-sex school | 0.01 | -0.67 to 0.69 | 0.97 |
| Faith school | -0.22 | -0.82 to 0.38 | 0.48 |
| if CM has longstanding illness | -0.08 | -0.7 to 0.54 | 0.80 |
| Parental Kessler score | 0.04 | -0.02 to 0.09 | 0.16 |
| Income | 0.13 | -0.34 to 0.59 | 0.59 |
| Urban classification | -0.08 | -0.68 to 0.53 | 0.8 |
| Short Moods and Feelings Questionnaire score | 0.02 | -0.03 to 0.07 | 0.45 |
| Total life satisfaction score | 0.04 | -0.01 to 0.08 | 0.11 |
| Safe for CM to walk/play etc within a mile/20 min from home | 0.43 | -0.40 to 1.26 | 0.31 |
| Has CMs school told you CM has special educational needs | 0.05 | -0.75 to 0.85 | 0.90 |
| Any religion | -0.24 | -0.71 to 0.24 | 0.33 |
| Non-white ethnicity | 0.43 | -0.16 to 1.03 | 0.15 |

**Table S9**: Full results from multivariable linear regression models for treatment effect heterogeneity of physical exercise at age 17 on psychological distress at age 17.

**REFERENCES**

Angold, A., Costello, E. J., Messer, S. C., & Pickles, A. (1995). Development of a short questionnaire for use in epidemiological studies of depression in children and adolescents. *International Journal of Methods in Psychiatric Research, 5*(4), 237-249.

Bannink, R., Pearce, A., & Hope, S. (2016). Family income and young adolescents’ perceived social position: associations with self-esteem and life satisfaction in the UK Millennium Cohort Study. *Archives of Disease in Childhood, 101*(10), 917-921.

Breiman, L. (2001). Random forests. *Machine Learning, 45*, 5-32.

Chen, T., & Guestrin, C. (2016). *Xgboost: A scalable tree boosting system.* Paper presented at the Proceedings of the 22nd ACM SIGKDD International Conference on Knowledge Discovery and Data Mining.

Friedman, J., Hastie, T., Tibshirani, R., Narasimhan, B., Tay, K., Simon, N., . . . Yang, J. (2020). glmnet: Lasso and Elastic-Net Regularized Generalized Linear Models, 2020. *R package version, 4*(2).

Funk, M. J., Westreich, D., Wiesen, C., Stürmer, T., Brookhart, M. A., & Davidian, M. (2011). Doubly robust estimation of causal effects. *American Journal of Epidemiology, 173*(7), 761-767.

Gruber, S., & Laan, M. v. d. (2012). tmle: An R Package for Targeted Maximum Likelihood Estimation. *Journal of Sstatistical Software, 51*(13), 1 - 35.

Ministry of Housing Communites & Local Government. (2020). English indices of deprivation. <https://www.gov.uk/government/collections/english-indices-of-deprivation>

Naimi, A. I., & Whitcomb, B. W. (2023). Defining and Identifying Average Treatment Effects. *American Journal of Epidemiology*. *5,* 192(5):685-687

Office for National Statistics. Rural/urban classifications. <https://www.ons.gov.uk/methodology/geography/geographicalproducts/ruralurbanclassifications>

R Core Team. (2019). R: A Language and Environment for Statistical Computing: R Foundation for Statistical Computing. <https://www.R-project.org/>

Rodrigues, D., Kreif, N., Lawrence-Jones, A., Barahona, M., & Mayer, E. (2022). Reflection on modern methods: constructing directed acyclic graphs (DAGs) with domain experts for health services research. *International journal of epidemiology, 51*(4), 1339-1348.

Rosenberg, M. (1965). Rosenberg self-esteem scale (RSE). *61*(52), 18.

Schuler, M. S., & Rose, S. (2017). Targeted maximum likelihood estimation for causal inference in observational studies. *American Journal of Epidemiology, 185*(1), 65-73.

Staley, K. (2013). There is no paradox with PPI in research. *Journal of Medical Ethics, 39*(3), 186-187.

StataCorp. (2021). Stata Statistical Software: Release 17. College Station, TX.

Tibshirani, J., Athey, S., Sverdruo, E., & Wager, S. (2022). grf: Generalized Random Forests. *R package version 2.2.1*.

Van der Laan, M. J., Polley, E. C., & Hubbard, A. E. (2007). Super learner. *Statistical Applications in Genetics and Molecular Biology, 6*(1).

Wager, S., & Athey, S. (2018). Estimation and inference of heterogeneous treatment effects using random forests. *Journal of the American Statistical Association, 113*(523), 1228-1242.
